# Curcumin on Camphor-Induced Chemical Burn on the Palate: Report of a Peculiar Self-Inflicted Case

**DOI:** 10.7759/cureus.20589

**Published:** 2021-12-21

**Authors:** Praveena Raman

**Affiliations:** 1 Oral Medicine and Radiology, Sathyabama Dental College and Hospital, Chennai, IND

**Keywords:** burns, camphor, chemical burn, curcuma longa, curcumin, non-suicidal self-injury, oral ulcer, palate, self-inflicted injury

## Abstract

Intraoral soft tissue injuries occur due to physical, chemical, or thermal agents, which may present as ulcerations, burns, desquamation, and gingival recession. Camphor is one such substance easily available in many Indian households and is not a very well-recognized potentially fatal toxic compound. Here, we report the first case of an intraoral soft-tissue burn in a geriatric female, as a result of direct injury to tissues due to the application of crushed camphor powder to manage tooth pain at home, which was successfully treated with 2% Curcuma longa. Health care professionals must be aware of the presentation and extent of injury that can be caused by camphor when placed on intraoral soft tissues. Awareness among the public and professionals must be created in order to avoid any potential mortality. The diagnostician must consider the possibility of a chemical agent, such as camphor, as a potential source of the oral mucosal injury.

## Introduction

Intraoral soft tissue injuries occur due to physical, chemical, or thermal agents because of iatrogenic, accidental, or factitious traumas, which may present as ulcerations, burns, desquamation, and gingival recession. The clinical presentation of such lesions might differ according to the composition of the causative agent, its pH, concentration, the quantity applied, the manner and duration of tissue contact, the extent of penetration into tissue, and the mechanism of action. Prolonged contact with the chemical agent can lead to epithelial necrosis and shedding [1-2].

Camphor is an easily available substance in many Indian households and is not a well-recognized, potentially fatal toxic compound. Camphor is a waxy, white, sublimating chemical derived from natural as well as synthetic sources and widely used in various communities worldwide for a number of medicinal, culinary, and religious reasons. It is also burnt as an offering to God in many religious communities [3].

“Curcumin” (diferuloylmethane) is the principal curcuminoid of the popular Indian spice “turmeric,” which is a member of the ginger family. Studies show that Curcumin is as powerful as an antioxidant, and it downregulates the expression of interleukin (IL)-6, tumor necrosis factor (TNF), and other chemokines. It is also been extensively investigated in various oral mucosal conditions like aphthous ulcers, oral lichen planus, oral submucous fibrosis, and other oral potentially malignant disorders [4-5]. Camphor-induced intraoral burns are rarely reported in the English literature

The purpose of this report is three-fold. First is the rarity of reporting a camphor-induced burn in a geriatric female. To our knowledge, we report the first case of camphor-induced intraoral burn treated successfully with Curcumin. The second is to highlight the effect of self-inflicted injury and finally to generate an awareness in clinicians on the possibility of camphor as a potent chemical in the causation of intraoral soft-tissue burns.

We believe that this knowledge will help clinicians have more empathetic and compassionate communication with geriatric patients with appropriate counseling for those who injure themselves [6].

## Case presentation

An 87-year-old female presented with tooth pain and burning sensation around the tooth and on the roof of the mouth for one day causing discomfort during speech and mastication. The history of the presenting illness revealed that she developed tooth pain while chanting, and she immediately managed this at home by placing crushed camphor around the painful tooth region and on the roof of her mouth. Also, she rinsed with warm water thinking that the pain will wash off in the camphor liquid by the time she completed her chanting, as she believed that God would cure her problems and sins. In a few minutes, she developed an intraoral burning sensation with redness. Psychosocial history revealed that the patient preferred only Ayurveda for all her medical problems and not allopathy due to a history of exfoliative dermatitis and Steven Johnson’s syndrome, followed by hospitalization on taking an analgesic during her childhood. No other relevant past medical history and medication history was reported.

**Figure 1:**
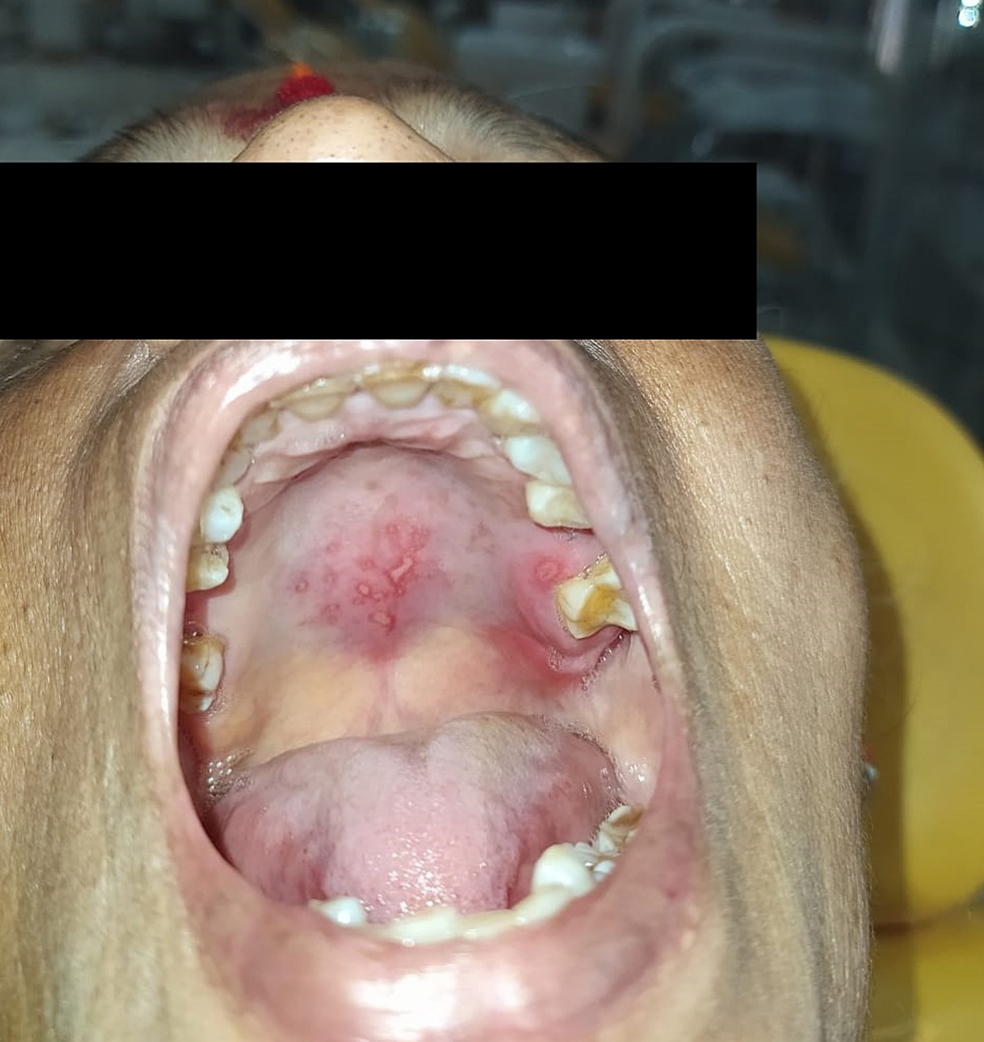
Pretreatment image of camphor-induced palatal burn and surrounding tooth 27

Intraoral examination revealed extensive ulcerations on the posterior central palatal region with erythematous borders, pseudomembrane, and yellow base and a single, well-defined ovoid ulcer on the palatal gingiva with respect to tooth 27 with surrounding erythema as shown in Figure 1.

Tooth 27 was Grade-I mobile with periodontal pocket and gingival recession without any carious lesion. Examination of the surrounding tissues and lymph nodes was non-contributory. A provisional diagnosis of Oral ulcers and a differential diagnosis (DD) of a chemical burn, Erythema multiforme (EM), and Herpes simplex virus (HSV; secondary) infection was made. Routine hematological investigations were within normal limits. Biochemical assay ruled out endocrinal abnormalities. Based on history, clinical presentation, and judgment by two oral medicine experts after excluding the DDs, the soft-tissue lesion, and the periodontal pain were finally diagnosed as "camphor-induced chemical burn on the palate" and "generalized chronic periodontitis," respectively.

Therapeutic intervention and instructions

The following interventions and instructions were given: Curnext oral gel 2%, Curcuma longa 10 mg, thrice daily for one week to 10 days; oral prophylaxis as per patient’s convenience; patient counseling to stop applying camphor inside the mouth; soft and bland diet until the ulcer healed.

Assessment of burn pain

The burn pain was assessed and evaluated based on a combination of the Numeric and Verbal pain rating scale from 0-5 cm, according to the National Initiative on Pain Control™ (NIPC™). Subjective pain (morning, lunch, and bedtime pain; 0-no pain, 1-mild, 2-moderate, 3-severe, 4-very severe, and 5-worst possible pain) was recorded in a log diary [7].

Adherence, adverse events, and tolerability

Intervention adherence was 94%, assessed via self-report and log diary. Curcumin was well-tolerated and our patient was satisfactory with no adverse effects.

Follow-up and outcome

**Figure 2:**
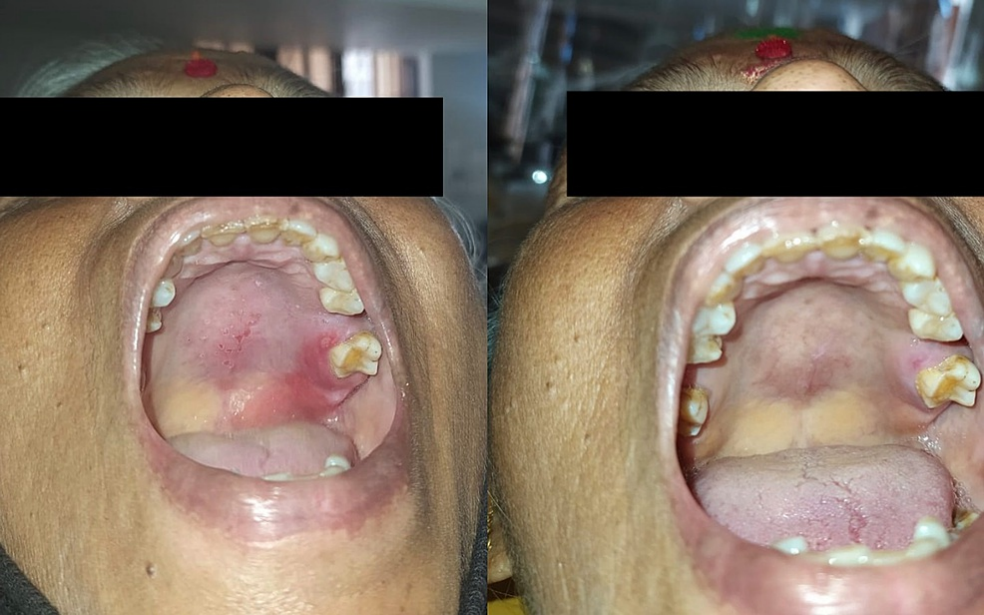
2a) Follow-up on Day 5 (left); Figure 2b: Post-treatment clinical presentation (right) 2a: Clinical presentation after treatment with 2% topical Curcuma longa; 2b: Palatal burn showing complete regression of the lesion

**Figure 3:**
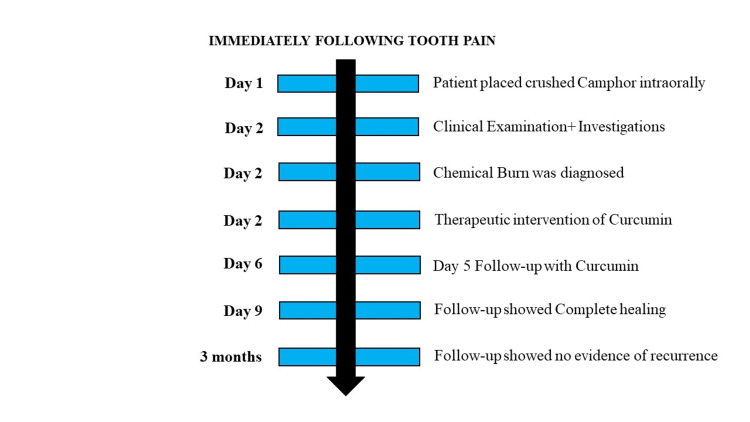
Case report timeline based on CARE guidelines CARE: CAse REport

Complete burn/ulcer healing, pain reduction, burn reduction, and re-epithelialization was the outcome. On Day 5, the patient had good resolution (Figure 2a). On Day 8, complete healing was noted (Figure 2b). The three-month follow‑up found the patient free of any oral ulcerations. The case report timeline is illustrated based on CARE (CAse REport) guidelines (Figure 3).

## Discussion

Burn injuries are a major healthcare issue. Any therapeutic model for symptomatic management to combat burns would benefit society in general. Non-suicidal self-injury (NSSI), also termed as 'self-harm, self-mutilation, or self-inflicted violence,' is a recently described entity in the Diagnostic and Statistical Manual of mental disorders 2013 (DSM V) [3]. According to Wilcox, 'self-injury can take various forms such as cutting, burning, or branding, hair pulling, picking at skin or scabs, headbanging, breaking bones, and hitting oneself.' Pain is defined as a psychic adjuvant of an imperative protective reflex or anything the patient feels hurts. Does pain have a role in an extraordinary state of consciousness? Does pain carry a cultural fact? Our patient had herself placed camphor inside her mouth. The decision to hurt involves a choice concerning consciousness itself.

Camphor, known as 1,7,7-Trimethylbicyclo[2.2.1]heptan-2-one is a cyclic ketone in the hydroaromatic terpene group. It is found in its natural form from the wood of the camphor laurel (Cinnamomum camphora), a large evergreen tree found in Asia. Synthetic camphor is obtained by distillation of turpentine. Once absorbed, it is oxygenated to produce the alcohol campherol which is then conjugated in the liver with glucuronic acid to become soluble in water. Most camphor is ultimately excreted in the urine. Camphor crosses the placental barrier, which accounts for its embryotoxic effects. In everyday usage, camphor is found in a variety of non-prescription products, either alone or in combination with other ingredients. It can also be purchased, particularly in shops providing alternative medications. Pharmaceutical products currently available on the market have camphor concentrations ranging from 0.5% to 10.8%, but it is also possible to find more concentrated products. Camphor oil preparations have been used both internally and externally in many countries, for a variety of ailments, ranging from respiratory problems to rheumatic pain. Camphor oil is applied to the skin as a rubefacient to promote circulation [8-9]. The mechanism by which camphor produces toxicity is unknown. Once absorbed, it is oxygenated to produce campherol. Within five to 15 minutes, patients commonly complain of mucus membrane irritation, nausea, vomiting, and abdominal pain. Chronic oral camphor has been reported to cause seizures and death as a form of NSSI [3,8].

Chemical burns are often localized and are rarely confined solely to the anatomic distribution of the masticatory mucosa, as demonstrated in our case. The contact of the oral mucosa with chemical substances usually develops as inflammation (redness) and ulcerations. Dentists may cause chemical burns by careless handling of various medications such as hydrogen peroxide, sodium hypochlorite solution, calcium hydroxide solution, and formocresol solution. The lesions produced vary according to the destructive properties and mode of application of the chemical agent.

To date, there is no one drug treatment for oral mucosal lesions. Steroids have always remained the mainstay for most physicians without considering the patient’s autonomy (patient’s choice/preference for therapeutic intervention). In our challenging case, Curcumin was chosen to meet our outcome, which is our strength. It is a proven wound healer in insect bites and chickenpox. It enhances burn healing by promoting re-epithelialization in rat models [10]. It inhibits lipid peroxidation, thereby reducing the rate of collagen synthesis. Agarwal et al. substantiated the clinical efficacy of Curcumin on burning sensation in oral submucous fibrosis [11]. Raman et al. reported that curcumin performed on par with steroids with respect to oral ulcer size, healing, and pain [4-5].

Our peculiar case of NSSI had limitations. First, restriction to use the most widely used drugs - routine steroidal or non-steroidal drugs and antibiotic mouth rinse. However, Curcumin was chosen based on a thorough literature search on its clinical efficacy on pain and burning sensation. Secondly, our patient could have missed the application of Curcumin; however, it is difficult to monitor.

## Conclusions

This peculiar clinical report illustrated a self-inflicted oral mucosal burn on the palate due to the placement of camphor, which was successfully managed with topical Curcumin. Burn injuries are a major healthcare issue. Burns can occur at home or at a dental office; however, they are very rarely reported in the literature. Early detection and management ensure a rapid cure, prevent further mucosal damage, and avoid any potential mortality. Diagnosticians must consider the possibility of camphor as a potent source of oral mucosal injury and must be aware of its oral presentation. Oral health care professionals must also consider using the herbal boon Curcumin for therapeutic intervention of such oral mucosal lesions.
